# *STK11*/LKB1 Loss in Cancer: From Developmental Constraint to Stress-Adapted Malignancy

**DOI:** 10.3390/cancers18172845

**Published:** 2026-09-03

**Authors:** Yu Kang, Yanhong Gao, Xiao-Yan Zhang, Hai-Ou Liu, Cong-Jian Xu, Yanying Huo

**Affiliations:** 1 Lab of Hereditary Gynecologic Oncology, School of Medicine, Westlake University, Hangzhou 310024, China; kangyu@westlake.edu.cn; 2 Department of Obstetric and Gynecology, Affiliated Hangzhou 1st People’s Hospital, School of Medicine, Westlake University, Hangzhou 310006, China; 3 Department of Clinical Laboratory, The First Medical Center of Chinese PLA General Hospital, Beijing 100853, China; gyh25cn@126.com; 4 Obstetrics & Gynecology Hospital of Fudan University, Shanghai Key Lab of Female Reproductive Endocrine Related Disease, Shanghai 200433, Chinaliuhaiou@fudan.edu.cn (H.-O.L.); 5 Department of Radiation Oncology, Rutgers Cancer Institute, 195 Little Albany Street, New Brunswick, NJ 08903, USA

**Keywords:** *STK11* (LKB1), Peutz–Jeghers syndrome, stress adaptation, tumor evolution, metabolic rewiring, redox tolerance, autophagy dependence, therapeutic resistance

## Abstract

Peutz–Jeghers syndrome (PJS) presents an apparent biological paradox: heterozygous germline pathogenic variants in *STK11* predispose to predominantly benign hamartomatous growth while conferring a markedly elevated lifetime risk of cancer, whereas somatic *STK11* inactivation in established tumors is frequently associated with aggressive progression and therapeutic resistance. *STK11* encodes liver kinase B1 (LKB1), a serine/threonine kinase that integrates metabolic, oxidative, architectural, and immune stress responses. Rather than acting solely as a direct brake on proliferation, LKB1 couples cellular growth, survival, and tissue organization to environmental fitness. We therefore propose a context-dependent stress adaptation framework in which impairment of *STK11*/LKB1 signaling relaxes stress-imposed constraints on cellular fitness, while the ultimate biological outcome is determined by allelic status, tissue context, and cooperating genetic alterations. In PJS, a heterozygous germline *STK11* pathogenic variant creates a constitutional cancer predisposed state in which one functional allele is initially retained, although subsequent loss or impairment of the remaining allele may occur during tumor evolution. In sporadic cancers, somatic *STK11* inactivation is often biallelic and frequently cooperates with alterations in *KRAS*, *KEAP1*, *TP53*, *NF1*, or PI3K-pathway genes. These genetic contexts can promote metabolic reprogramming, redox adaptation, autophagy dependence, immune exclusion, cellular plasticity, and therapeutic resistance, with the strongest mechanistic and clinical evidence currently derived from lung adenocarcinoma (LUAD). Within this framework, enhanced persistence under metabolic, oxidative, immune, and therapy-induced stress does not exclude proliferative effects of STK11 loss but provides a permissive background upon which cooperating oncogenic programs can drive clonal expansion and malignant progression. Stress adaptation creates dependencies on interconnected buffering systems, including antioxidant defenses, autophagy, metabolic plasticity, and ferroptosis suppression. Therapeutic strategies that simultaneously disrupt multiple compensatory pathways may therefore exceed tumor adaptive capacity, convert stress tolerance into therapeutic vulnerability, and provide a rational framework for treating LKB1-*deficient* tumors and other stress-adapted cancers.

## Simple Summary

The *STK11* gene encodes the serine/threonine kinase LKB1, which helps cells respond to metabolic, oxidative, and environmental stress. In Peutz–Jeghers syndrome, a heterozygous germline pathogenic variant in the *STK11* gene predisposes individuals to hamartomatous polyp formation and substantially increases their lifetime risk of cancer. In sporadic cancers, inactivation of *STK11* and the resulting loss of LKB1 function, together with additional oncogenic alterations, enables tumor cells to survive conditions that would normally restrict growth. This adaptability can promote metabolic reprogramming, immune evasion, and treatment resistance—particularly in lung cancer. However, it also creates dependencies on cytoprotective processes such as antioxidant defenses and cellular recycling. Simultaneously targeting multiple components of these systems may therefore improve therapeutic outcomes in LKB1-deficient cancers more broadly.

## 1. Introduction

Cellular survival under stress is a fundamental requirement for tissue homeostasis and disease progression. In multicellular organisms, cells are continuously exposed to fluctuating metabolic, oxidative, and inflammatory conditions and must integrate these inputs to direct whether to adapt, arrest, or undergo cell death. Dysregulation of stress-integration systems is increasingly recognized as a central driver of cancer evolution, shaping tumor initiation, therapeutic resistance, and immune interactions [1,2,3].

Among the core regulators of cellular stress integration is LKB1, a serine/threonine kinase encoded by the *STK11* gene. Heterozygous germline pathogenic variants in *STK11* cause Peutz–Jeghers syndrome (PJS), a hereditary disorder characterized by hamartomatous gastrointestinal polyps, mucocutaneous pigmentation, and elevated cancer risk [4,5,6]. LKB1 is best known as an upstream kinase that activates AMP-activated protein kinase (AMPK) and multiple AMPK-related kinases, thereby coupling cellular energy status to growth control, polarity regulation, and metabolic adaptation [7,8]. Through these pathways, LKB1 occupies a central position at the intersection of metabolism, spatial organization, and cellular stress response.

Inactivation of *STK11* and the consequent loss of LKB1 function have been implicated in diverse biological processes spanning development, epithelial organization, metabolic regulation and tumor suppression [9,10]. Notably, heterozygous germline pathogenic variants and somatic inactivation of *STK11* can produce distinct biological outcomes. In PJS, heterozygous germline pathogenic variants in *STK11* give rise to predominantly benign, noninvasive hamartomatous polyps that generally exhibit indolent behavior [11,12]. However, PJS is also associated with a markedly elevated lifetime risk of malignancy across multiple organ systems [13,14]. Importantly, these malignancies do not arise exclusively from hamartomatous polyps. Although a subset of gastrointestinal cancers may develop within or adjacent to these lesions, many—particularly those arising in extraintestinal organs, such as the pancreas, breast, and gynecologic organs—develop independently, reflecting the broader, tissue-wide cancer predisposition created by constitutional *STK11* haploinsufficiency. In contrast, somatic inactivation of *STK11*, which is often biallelic, is frequently observed in aggressive human cancers, most prominently LUAD, where it is associated with poor prognosis and resistance to multiple therapeutic modalities [15,16,17]. This apparent germline–somatic paradox suggests that the biological consequences of *STK11* alteration cannot be understood from mutation status alone but must be interpreted in relation to allelic dosage, tissue context, and the cooperating genetic landscape.

This divergence raises a fundamental conceptual question: how does impairment of LKB1-mediated stress integration alter the relationship among proliferation, survival, tissue organization, and environmental fitness, and why do these consequences differ between germline and somatic contexts? LKB1 has tumor suppressive functions in restraining anabolic growth and cell cycle progression [7,8,18,19]; however, these functions coexist with broader roles in energy sensing, redox homeostasis, autophagy, polarity, and stress-responsive survival. We therefore propose that *STK11* loss should not be viewed solely through a proliferation-centered framework. Rather, impairment of LKB1 signaling can relax stress-imposed constraints on cellular fitness, creating context-dependent opportunities for persistence, adaptation, and subsequent clonal expansion. Importantly, this model does not exclude direct or indirect effects of *STK11* loss on proliferation; instead, it emphasizes that proliferative advantage and stress tolerance can cooperate during tumor evolution, with their relative contributions determined by allelic status, tissue lineage, microenvironmental pressure, and cooperating oncogenic alterations.

Understanding why heterozygous germline pathogenic variants in *STK11* create a constrained yet cancer-prone state, whereas somatic *STK11* inactivation can promote aggressive, therapy-resistant disease, is therefore essential for identifying actionable vulnerabilities in *STK11* altered cancers. This divergence reflects not only differences in allelic status but also tissue context and cooperating genetic events. In PJS, a heterozygous germline *STK11* pathogenic variant creates a constitutional background in which one functional allele is retained. Subsequent tumor development or malignant progression may be facilitated by additional somatic events, including loss of heterozygosity or other mechanisms that impair the remaining allele, although complete biallelic inactivation is not uniformly observed in all PJS-associated lesions. In sporadic cancers, by contrast, somatic *STK11* inactivation is often biallelic, resulting in profound or complete loss of LKB1 function within tumor cells. Importantly, although PJS is characterized by predominantly benign hamartomatous lesions, it confers a markedly elevated lifetime risk of malignancy across multiple organ systems, underscoring that constrained growth at the tissue level can coexist with substantial cancer susceptibility at the organismal level. These observations further indicate that “*STK11* alteration” encompasses biologically distinct states—from constitutional heterozygosity and dosage reduction to acquired biallelic inactivation—and that these states should not be assumed to have equivalent functional consequences. In this Review, we analyze the biological functions of LKB1 and the consequences of *STK11* alteration across germline and somatic contexts, with particular emphasis on the maintenance of cellular equilibrium and the regulation of stress adaptation. We first summarize the molecular functions of LKB1 in metabolic and polarity signaling, then consider PJS as a model of constrained but cancer-prone tissue growth, followed by an analysis of somatic *STK11* loss in sporadic cancers. We subsequently develop a stress-adaptation framework to examine how impaired LKB1 signaling may facilitate adaptive persistence and interact with cooperating oncogenic programs during malignant progression. Because the mechanistic and clinical evidence is currently most developed in LUAD, we use lung cancer as the principal model while incorporating evidence from other tumor types where available. Finally, we explore therapeutic strategies aimed at collapsing interconnected stress-buffering systems in LKB1-deficient tumors and consider the extent to which this framework may be generalizable to other stress-adapted cancers.

## 2. STK11/LKB1 as a Regulator of Cellular Equilibrium

*STK11* was originally identified as the gene responsible for PJS through the discovery of truncating germline pathogenic variants in a gene encoding a serine/threonine kinase and located on chromosome 19p13.3 [5,6]. These foundational studies established *STK11* as a tumor-predisposition gene encoding the protein kinase LKB1, implicating disruption of core regulatory processes governing cellular organization and homeostasis, rather than the direct activation of mitogenic signaling, in tumor predisposition [4,5,6].

A hamartoma–adenoma–carcinoma sequence has been proposed for a subset of PJS-associated gastrointestinal malignancies, in which aberrant but initially constrained tissue growth may progressively evolve toward malignancy [4]. However, this sequence is not universal, and many PJS-associated cancers arise independently of hamartomatous polyps [20,21,22]. Nevertheless, this model suggests that partial or complete loss of LKB1 function does not necessarily trigger immediate, uncontrolled proliferation but instead perturbs a regulated equilibrium that normally balances growth, differentiation, and tissue architecture. Importantly, this interpretation does not imply that LKB1 lacks direct growth-regulatory functions; rather, it places these functions within a broader homeostatic network in which metabolic fitness, stress tolerance, differentiation, and tissue organization collectively determine whether altered cells remain constrained or progress toward malignancy. This concept of equilibrium disruption, rather than proliferative deregulation alone, provides a critical framework for understanding the diverse biological consequences of *STK11* alteration and LKB1 dysfunction across germline and somatic contexts.

### 2.1. Metabolic Equilibrium: Energy and Stress Constraint

At the molecular level, LKB1 functions as a central upstream kinase in the cellular energy-stress response, activating AMP-activated protein kinase (AMPK)—a principal sensor of cellular energy status—by phosphorylating Thr172 within the activation loop of its catalytic α subunit. This phosphorylation enables AMPK-dependent metabolic checkpoint control under conditions of energetic insufficiency. Through activation of AMPK and a broader family of AMPK-related kinases, LKB1 couples metabolic stress to growth restraint and context-dependent survival decisions [7]. In this context, proliferation and biosynthetic programs remain tightly constrained by cellular energy availability, ensuring that growth is contingent on adequate metabolic support [8].

Importantly, the biological role of AMPK in cancer is context-dependent and cannot be reduced to a uniformly tumor-suppressive output. During tumor initiation or under conditions of excessive anabolic signaling, LKB1–AMPK activation can restrain growth by suppressing energy-consuming biosynthetic programs and limiting mTOR-dependent signaling [7,23]. In established tumors, however, AMPK activation can also support cellular fitness during metabolic stress by restoring energy balance, promoting catabolic metabolism and autophagy, and limiting oxidative damage [7,23]. Thus, the LKB1–AMPK axis can simultaneously impose growth restraint and support stress survival, with the balance between these functions determined by cellular state and environmental context. This duality is particularly relevant to LKB1-deficient cancers, in which loss of canonical LKB1–AMPK signaling may remove growth constraints while also generating energetic and oxidative liabilities that select for alternative compensatory survival programs.

In cancer, *STK11* inactivation and the resulting impairment of LKB1 signaling weaken LKB1-dependent metabolic checkpoint control, allowing cells to continue growth or survive despite energetic imbalance in permissive genetic and environmental contexts. This uncoupling of metabolic stress from growth control can promote anoikis resistance, metastatic competence, and the selection of stress-adapted cellular states [24]. However, *STK11* loss should not be interpreted as conferring intrinsic or generalized metabolic robustness. Instead, impaired LKB1 signaling can generate substantial energetic and oxidative stress, creating selective pressure for cells capable of engaging alternative metabolic and cytoprotective programs. Rather than conferring generalized metabolic robustness, the resulting phenotype therefore reflects conditional adaptation to specific stresses and may simultaneously expose metabolic dependencies that can be therapeutically exploited.

Beyond energy sensing, LKB1 coordinates redox homeostasis and stress resilience. The SIRT1–LKB1–AMPK network is not strictly linear but is influenced by upstream and feedback regulatory inputs; for example, SIRT1-mediated deacetylation of LKB1 can enhance its kinase activity, thereby promoting downstream AMPK activation [25]. Through AMPK-dependent and related stress-response pathways, LKB1 contributes to autophagic regulation and metabolic homeostasis under cellular stress [26]. In *KRAS*-mutant NSCLC models, restoration of LKB1 enhanced AMPK activation, ULK1 Ser555 phosphorylation, and autophagic activity, whereas LKB1 loss reduced this adaptive autophagic response; importantly, this LKB1–AMPK–autophagy program could protect stressed tumor cells from therapy-induced senescence [26]. These findings illustrate that LKB1 itself can support adaptive survival under specific stress conditions and that LKB1 loss does not intrinsically confer enhanced stress tolerance. Notably, LKB1 also engages pathways outside canonical AMPK signaling, including p53-dependent induction of p21 and cell-cycle arrest [18,19]. LKB1 can additionally promote p53-dependent apoptosis in response to selected cellular stresses, although during energetic stress, the LKB1–AMPK pathway can instead support cellular survival by promoting metabolic adaptation and limiting excessive stress induced damage [27,28]. These apparently divergent outputs illustrate an important feature of LKB1 biology: its tumor-suppressive activity does not arise from a single obligatory cellular outcome, but from context-dependent coordination of growth restraint, stress adaptation, cell-cycle arrest, and, when stress becomes incompatible with homeostasis, cell death. These stress responses are functionally interconnected: impaired energy sensing can amplify redox stress, which in turn may increase reliance on compensatory autophagy and antioxidant programs. Thus, LKB1 integrates metabolic and redox inputs into a unified stress-response architecture rather than regulating these processes in isolation.

**Figure 1:**
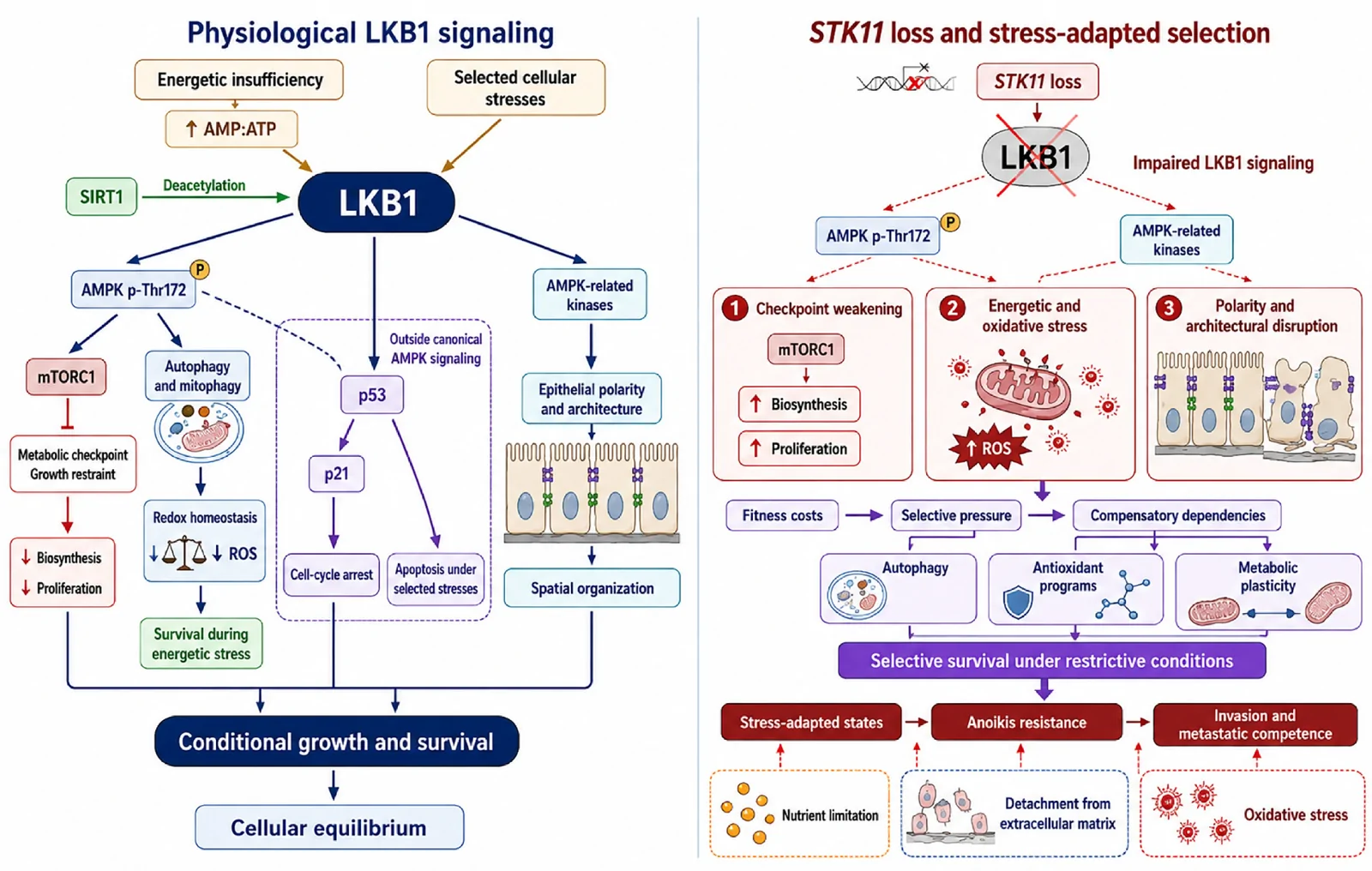
**LKB1 integrates metabolic and cellular stress responses.** Under physiological conditions, LKB1 activates AMPK and AMPK-related kinases to coordinate metabolic restraint, redox homeostasis, autophagy, cell-cycle control, and epithelial polarity. Loss of *STK11* impairs LKB1 signaling, weakening these constraints and imposing energetic, oxidative, and architectural stress. Subsequent selection for compensatory autophagy, antioxidant defenses, and metabolic plasticity promotes survival under restrictive conditions and contributes to anoikis resistance, invasion, and metastatic competence. Solid arrows indicate established regulatory or functional relationships, dashed arrows indicate impaired signaling following *STK11* loss, and T-bars indicate inhibitory effects. Red arrows and symbols denote changes or consequences associated with *STK11* loss, whereas blue/green elements represent physiological LKB1-associated signaling and purple elements indicate stress-adaptive or compensatory responses. “P” denotes phosphorylation.

Together, these functions position LKB1 not simply as a component of a linear signaling pathway but as an integrative regulator of cellular equilibrium [7]. By coordinating energy sensing, redox homeostasis, autophagic flux, and epithelial polarity through AMPK-dependent and AMPK-related kinase pathways, LKB1 imposes conditional constraints that couple cellular behavior to environmental fitness [23,29,30]. Under physiological conditions, these constraints ensure that proliferation, survival, and spatial organization remain contingent on adequate metabolic and structural support. *STK11* loss and the resulting impairment of LKB1 signaling weaken this coupling. Rather than directly conferring generalized stress resistance, this disruption creates selective pressure for compensatory cellular states capable of tolerating energetic stress, oxidative stress, and architectural disruption while maintaining survival under otherwise restrictive conditions [30,31]. Accordingly, the stress-adapted phenotype discussed in this Review should be understood not as a direct and universal consequence of *STK11* loss but as an evolved state that can emerge when LKB1-deficient cells successfully compensate for the metabolic and redox liabilities created by loss of normal stress integration (Figure 1).

### 2.2. Spatial and Polarity Equilibrium: Architectural Constraint

Metabolic regulation and polarity control are not independent outputs of LKB1 activity but mechanistically integrated dimensions of cellular homeostasis. Maintenance of epithelial polarity imposes substantial energetic and cytoskeletal demands, whereas metabolic stress can directly affect cytoskeletal dynamics, membrane trafficking, and junctional stability [32]. Through AMPK-related kinases and polarity-associated protein complexes, LKB1 couples metabolic status to spatial organization, thereby preserving epithelial integrity, barrier function, and tissue architecture [29,33]. Disruption of this integration increases cellular plasticity, weakens architectural constraints, and can facilitate adaptation to altered metabolic and microenvironmental conditions without necessarily producing malignant transformation [32].

At the molecular level, LKB1 regulates epithelial polarity and tissue organization through its kinase activity and controlled subcellular localization. Functional analyses of PJS-associated *STK11* variants indicate that the C-terminal region of LKB1 is important for both AMPK signaling and polarity regulation [34]. LKB1 forms a heterotrimeric complex with the pseudokinase STRAD and the scaffolding protein MO25, which stabilizes and activates LKB1, promotes its cytoplasmic localization, and enables downstream polarity-associated signaling [33,35]. Through regulation of Cdc42-dependent cytoskeletal organization, LKB1 supports polarized cellular architecture and spatial signaling fidelity, thereby constraining cellular plasticity and maintaining the structural organization within which growth and survival signals are normally interpreted [36].

These homeostatic functions extend beyond individual cells to higher-order tissue architecture and spatial organization [7]. By regulating cell size, polarity, adhesion, and stress-adaptation programs, LKB1 helps maintain organized epithelial structure and restrict the emergence of spatially disordered growth states [29,33]. Loss of *STK11* and the resulting impairment of LKB1 signaling can progressively weaken tissue-level organization, allowing cells to remain viable while becoming increasingly uncoupled from normal architectural and microenvironmental constraints [6,29]. Importantly, architectural disruption should be viewed as a permissive rather than uniformly transforming consequence of LKB1 deficiency. Loss of polarity and altered adhesion can expand cellular plasticity and increase tolerance of spatial displacement, but whether these changes progress to invasive malignancy depends on allelic dosage, lineage-specific programs, microenvironmental conditions, and cooperating oncogenic alterations. Disruption of LKB1-dependent homeostasis may therefore create a permissive tissue state from which divergent biological phenotypes emerge under distinct genetic and environmental pressures [31,37].

Consistent with this model, *STK11* deficiency and loss of LKB1 function disrupt polarity and tissue organization across diverse biological systems, affecting germline stem-cell quiescence [38], epithelial integrity and polarity signaling [29], Sertoli cell architecture [39], pancreatic acinar organization [40], and neuronal and glial polarity [41,42]. At the tissue level, loss of *STK11* can also alter adhesion signaling and extracellular-matrix organization, promoting collagen remodeling and invasive behavior [43]. Together, these observations indicate that disruption of LKB1-dependent spatial regulation is a conserved biological consequence across multiple cellular and tissue contexts, although its phenotypic outcome is strongly context-dependent. Studies in Drosophila further underscore the evolutionarily conserved role of LKB1 in maintaining spatial and functional cellular equilibrium [44].

Taken together, the metabolic and architectural functions of LKB1 define complementary dimensions of cellular equilibrium: metabolic signaling couples growth and survival to energetic fitness, whereas polarity and adhesion couple cellular behavior to spatial and tissue-level constraints. *STK11* loss can weaken both forms of coupling, increasing the range of metabolic and architectural conditions under which altered cells can persist. However, this permissive state is not synonymous with malignancy. Rather, it provides a context in which subsequent genetic, cellular, and microenvironmental events can determine whether altered tissue remains constrained, as frequently observed in PJS-associated hamartomas, or evolves toward invasive and stress adapted cancer.

## 3. Germline STK11 Alteration: Peutz–Jeghers Syndrome as a Model of Constrained Growth

PJS is most commonly caused by heterozygous germline loss-of-function pathogenic variants in *STK11* and is clinically characterized by mucocutaneous pigmentation and multifocal gastrointestinal hamartomatous polyps [45,46]. This constitutional heterozygous state should be distinguished from complete *STK11* loss: one wild-type allele is initially retained, although reduced *STK11* dosage can itself perturb tissue homeostasis in a context-dependent manner. At the genetic level, *STK11* functions as a context-dependent, dosage-sensitive tumor suppressor: a germline pathogenic variant establishes a cancer-predisposed background, whereas somatic loss of the remaining wild-type allele—frequently through loss of heterozygosity or another inactivating event—may provide a second hit during neoplastic progression [47,48]. However, biallelic inactivation is not uniformly demonstrated in all PJS-associated lesions, and heterozygous *STK11* deficiency can itself produce biological phenotypes consistent with haploinsufficiency in experimental systems [49,50]. Thus, germline heterozygosity, functional haploinsufficiency, and acquired biallelic loss represent related but biologically distinct states and should not be used interchangeably. Complete *STK11* loss can produce distinct biological consequences depending on cellular and tissue context, ranging from altered differentiation and epithelial polarity defects to tissue disorganization, aberrant growth, and context-dependent tumorigenesis [29,39,40,41,50,51].

Despite the presence of a tumor suppressor gene defect, PJS represents a biological state in which growth regulation is disrupted but not completely uncoupled from tissue-level organizational constraints. Patients develop extensive and recurrent tissue overgrowth, yet the dominant phenotype is hamartomatous expansion rather than overtly aggressive malignancy. Importantly, cancer susceptibility is markedly elevated at the organismal level, with cumulative lifetime risk approaching 90% across multiple organ systems [13], whereas the malignant potential of individual lesions remains variable and highly context-dependent. This apparent dissociation highlights a fundamental distinction between permissive tissue overgrowth and fully malignant transformation.

At the morphological level, PJS polyps retain relatively organized epithelial–stromal architecture, consistent with dysregulated yet differentiated tissue compartments, rather than invasive, dedifferentiated carcinoma [48]. Arborizing smooth muscle extension and largely preserved epithelial organization are characteristic features, indicating that higher-order tissue architecture remains substantially intact despite aberrant growth. Clinically, most individuals experience decades of recurrent polyp burden—manifesting as bleeding, anemia, or intussusception—while individual lesions typically remain benign and do not uniformly progress through a hamartoma–dysplasia–carcinoma sequence [47,52,53]. Instead, malignancies in PJS arise through diverse, tissue-specific evolutionary pathways, many of which are not directly derived from pre-existing hamartomatous polyps [47]. Accordingly, the high cancer risk of PJS should not be interpreted as evidence that hamartomatous polyps are obligate premalignant lesions; rather, constitutional *STK11* alteration creates a broader tissue-wide susceptibility within which malignant evolution can occur through multiple routes.

For example, glandular lesions arising in specialized epithelial contexts can undergo progression from hyperplasia to invasive carcinoma. This pattern is illustrated by cervical glandular neoplasia, in which precursor lesions such as lobular endocervical glandular hyperplasia are frequently associated with *STK11* alterations and may progress to gastric-type adenocarcinoma [54]. Consistent with this, germline *STK11* deficiency in PJS is associated with an increased risk of diverse gynecologic malignancies, including cervical glandular tumors [13], reflecting tissue-specific vulnerabilities linked to the developmental and functional context of LKB1 signaling [14]. These observations illustrate that the consequences of *STK11* deficiency are strongly lineage dependent: a similar initiating genetic defect may remain morphologically constrained in one tissue yet create a substantially more permissive substrate for neoplastic progression in another.

Together, these features indicate that heterozygous germline *STK11* alteration weakens growth restraint while allowing substantial preservation of tissue architecture and differentiation in many PJS-associated lesions, resulting in a state of constrained yet permissive growth in which malignant transformation remains context-dependent and proceeds through multiple, lineage-specific evolutionary trajectories rather than a uniform stepwise process. Genetically engineered mouse models further reinforce this constrained-growth paradigm. *Stk11* heterozygous mice develop spontaneous gastrointestinal hamartomas that closely recapitulate human PJS, demonstrating that partial *Stk11* loss is sufficient to induce aberrant tissue expansion in a context-dependent manner but, within the intestinal setting, is generally insufficient to drive overt malignant transformation [49,50].

Importantly, the biological consequences of *STK11* alteration are not uniform across tissues. Whereas gastrointestinal epithelial lesions typically remain relatively constrained, *STK11* deficiency within specific stromal or mesenchymal compartments—including Müllerian-derived tissues—can promote hyperplasia, neoplasia, and tumorigenesis [51]. Experimental models further show that the phenotype produced by *Stk11* deficiency depends on both cellular compartment and cooperating genetic context [50,55]. These findings argue against a simple linear relationship between the extent of *STK11* loss and malignant potential; instead, allelic state interacts with lineage, developmental program, and tissue architecture to determine phenotypic outcome.

The transition from a PJS-associated constrained state to malignancy therefore requires consideration of more than *STK11* status alone. A constitutional heterozygous pathogenic variant establishes a field of increased susceptibility, but subsequent evolution can involve several non-mutually exclusive processes. First, somatic impairment or loss of the remaining wild-type *STK11* allele can further reduce LKB1 activity in selected clones [20,56,57]. Second, cooperating oncogenic or tumor-suppressive alterations can provide growth, survival, or genomic-fitness advantages that are not supplied by partial *STK11* deficiency alone [50]. Third, lineage-specific differentiation programs and stromal organization can determine whether altered cells remain architecturally constrained or acquire greater plasticity. Finally, microenvironmental pressures including metabolic limitation, oxidative stress, inflammation, and immune surveillance can select for clones capable of persisting despite progressive loss of normal homeostatic control.

These processes suggest that *STK11* deficiency may lower the evolutionary barrier to malignancy without prescribing a single route of progression. In this model, loss of LKB1-dependent homeostatic control expands the range of cellular states that can persist, while second-hit events, cooperating genetic alterations, and tissue-specific selection determine which of these states undergo clonal expansion and malignant escape. Importantly, the relative contribution and temporal order of these events are likely to differ among tissues and remain incompletely defined in many PJS-associated cancers. The proposed sequence should therefore be viewed as an integrative framework rather than a universal linear progression model. The genetic and functional heterogeneity of *STK11* alteration across these contexts is summarized in Table 1.

Thus, PJS can be viewed as a model of partial tissue homeostatic disruption in which the coupling between growth and environmental restraint is weakened, permitting tissue expansion under conditions that would normally restrict proliferation, while higher-order organizational constraints remain relatively preserved [48,50,55]. In this intermediate state, epithelial cells remain permissive for expansion yet constrained by retained tissue architecture, differentiation programs, and spatial organization, thereby limiting-but not eliminating-the potential for lesion-level malignant transformation [48,50,55]. Importantly, the markedly elevated lifetime cancer risk associated with PJS reflects a systemic, multi-organ susceptibility to tumorigenesis rather than an intrinsic propensity of individual hamartomatous polyps to undergo malignant transformation. Consistent with this, PJS-associated cancers arise across both gastrointestinal and extra-intestinal tissues and are not necessarily derived from pre-existing hamartomatous lesions, which themselves typically remain benign and do not constitute obligate precursors to carcinoma [13,60].

Collectively, clinical observations and genetically engineered models support a framework in which heterozygous germline *STK11* alteration establishes a dosage-sensitive, cancer-predisposed state rather than an immediately malignant phenotype. Progression can involve further loss of LKB1 function together with tissue-specific and cooperating genetic events that progressively relax architectural, metabolic, and microenvironmental constraints [47,48,50]. PJS therefore provides a useful biological model for distinguishing tumor predisposition from tumor execution: *STK11* alteration creates permissiveness, whereas the route from permissiveness to invasive malignancy is determined by subsequent evolutionary context.

## 4. Somatic STK11 Loss in Cancer: Disruption of Equilibrium

**Figure 2:**
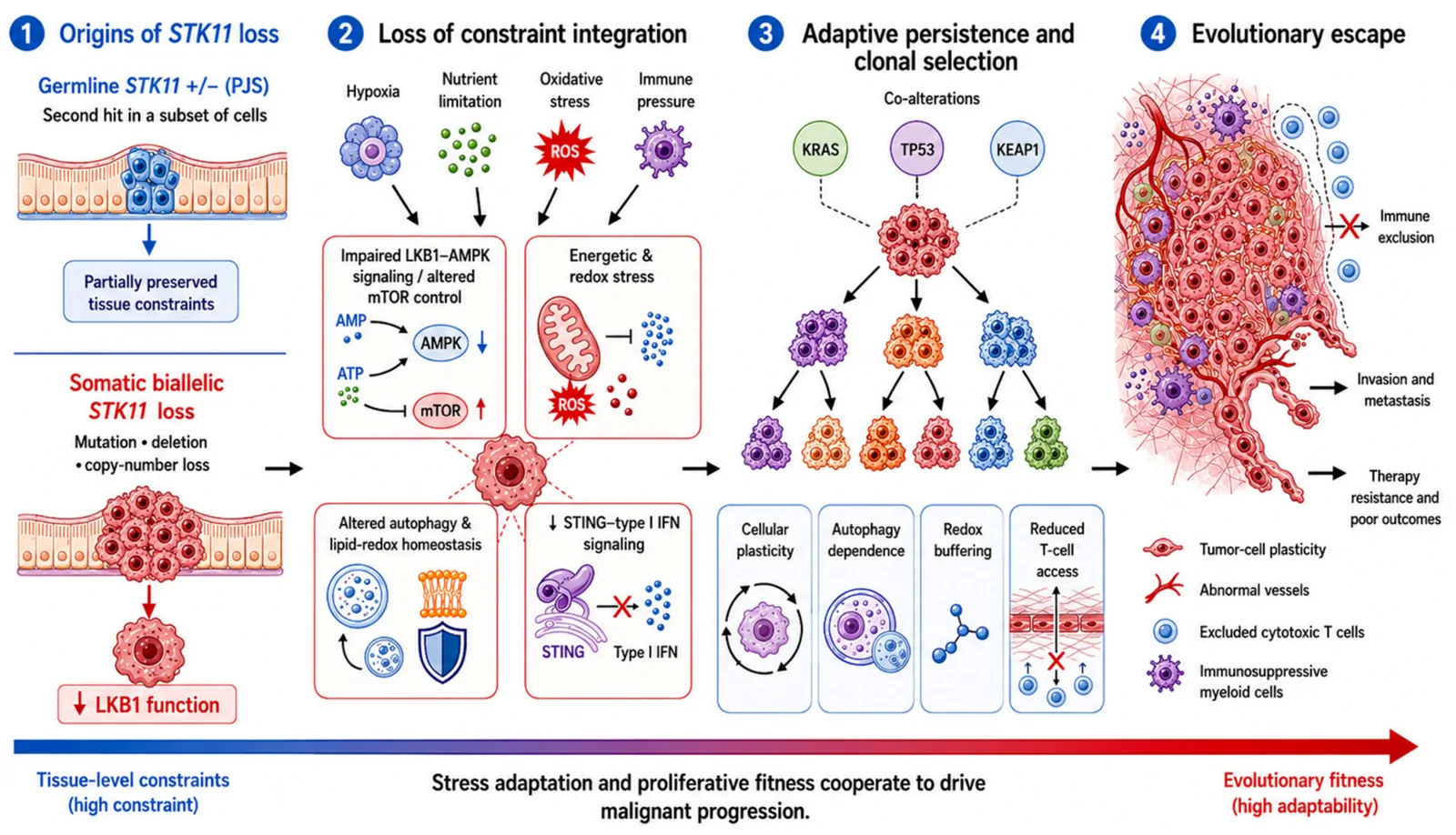
***STK11* loss, adaptive persistence, and evolutionary escape.** In Peutz–Jeghers syndrome (PJS), a germline heterozygous *STK11* alteration initially retains one functional allele, and a second hit occurs only in a subset of cells, allowing tissue-level constraints to remain partially preserved. In contrast, somatic biallelic *STK11* inactivation causes marked loss of LKB1 function. Under metabolic, oxidative, and immune stress, LKB1 deficiency disrupts stress integration, resulting in impaired LKB1–AMPK signaling, altered mTOR control, energetic and redox stress, altered cellular quality control, and impaired STING–type I interferon signaling. Importantly, LKB1 deficiency does not itself confer generalized stress resistance; rather, these liabilities create selective pressures that favor compensatory cellular states. Cooperating alterations in *KRAS*, *TP53*, or *KEAP1* further shape clonal selection and adaptive persistence through context-dependent programs involving cellular plasticity, autophagy, redox buffering, and immune evasion. These adaptations can promote evolutionary escape, invasion and metastasis, and therapeutic resistance, with stress adaptation and proliferative fitness cooperating to drive malignant progression. Arrows indicate directional relationships or progression; upward and downward arrows indicate increased and decreased activity, respectively, and red X symbols indicate inhibition or exclusion. Colors are used schematically to distinguish functional states, cellular phenotypes, and clonally selected tumor-cell populations and do not represent quantitative measurements unless otherwise indicated.

In contrast to the constrained aberrant growth observed in the constitutional heterozygous state of PJS, somatic inactivation of *STK11* in sporadic cancers represents a distinct genetic and biological state shaped by differences in allelic status, tissue context, and cooperating genetic events. As discussed above, a heterozygous germline STK11 pathogenic variant in PJS initially retains one functional allele, and additional loss or impairment of the remaining allele occurs in only a subset of lesions. By contrast, somatic *STK11* inactivation in sporadic tumors can involve mutation, deletion, copy-number loss, or combinations of these events, and biallelic inactivation can result in profound or complete loss of LKB1 function within tumor cells [9,14,59]. Across multiple tumor types, including lung adenocarcinoma, pancreatic ductal adenocarcinoma, melanoma, and gynecologic malignancies—*STK11* inactivation is associated with aggressive biological behavior, metastatic progression, therapeutic resistance, and adverse clinical outcomes [17,61,62,63,64,65,66,67]. However, the strength and mechanistic depth of these associations vary substantially among tumor types, with the most extensive experimental and clinical evidence currently derived from LUAD. In these contexts, loss of LKB1 activity can disrupt the integration of metabolic stress responses, redox homeostasis, and tissue-organizational programs [29,30,68]. Rather than producing a uniform phenotype, this disruption creates a context-dependent state in which tumor evolution is increasingly shaped by the capacity of individual clones to compensate for the metabolic, oxidative, architectural, and immune pressures associated with LKB1 deficiency (Figure 2).

Genomic analyses demonstrate that *STK11* alterations frequently coexist with alterations in *KRAS*, *TP53*, and *KEAP1*, particularly in LUAD, defining recurrent co-mutational configurations that shape tumor fitness, clonal selection, immune phenotype, and therapeutic vulnerability [9,61,69,70,71]. These cooperating alterations should not be regarded as interchangeable modifiers of *STK11* loss, because they affect distinct biological constraints. *KRAS* activation provides sustained mitogenic and anabolic signaling that can drive proliferative expansion in a background in which LKB1-dependent metabolic checkpoints are impaired [61,69,71]. KEAP1 loss activates NRF2-dependent antioxidant and detoxification programs, enhancing redox buffering and metabolic flexibility and thereby helping cells tolerate oxidative and metabolic liabilities associated with *STK11* deficiency [70,72,73,74,75]. *TP53* loss or dysfunction can relax p53-dependent cell-cycle checkpoints, senescence, and apoptotic responses to cellular stress, potentially increasing the tolerance of genomically or metabolically stressed cells to otherwise growth-limiting conditions [76,77]. Thus, different co-mutational configurations can supply complementary capabilities—proliferative drive, redox buffering, or checkpoint escape—that interact with LKB1 deficiency to generate biologically distinct evolutionary trajectories.

This distinction is particularly evident in *KRAS*-mutant LUAD. *KRAS/STK11* and *KRAS/TP53* tumors represent molecularly and immunologically distinct disease states rather than equivalent *KRAS*-driven cancers [69,70,71]. *KRAS/STK11* tumors are commonly associated with an immune-cold or immune-excluded phenotype, whereas *KRAS/TP53* tumors can exhibit greater inflammatory signaling and immune infiltration. Additional KEAP1 inactivation within the LKB1-deficient background can further reinforce antioxidant and metabolic adaptation and is associated with particularly adverse clinical behavior and therapeutic resistance [61,67,72,73,74,75,78,79]. These observations support a modular model in which the phenotype attributed to “*STK11*-mutant cancer” is constructed jointly by LKB1 deficiency and the specific cooperating genetic landscape rather than by *STK11* status alone.

Accordingly, *STK11* loss is better viewed as a context-setting alteration than as an isolated determinant of tumor behavior. LKB1 regulates how cells respond to metabolic, immune, and structural pressures [9,37,71,80,81]. Loss of LKB1 disrupts AMPK–mTOR signaling and associated metabolic stress-response programs, creating energetic and redox disequilibrium while relaxing growth control [7,23,31,59,74,82,83]. Cells that successfully persist in this setting can be selected for compensatory metabolic programs that support survival during energetic stress, hypoxia, and nutrient limitation. Thus, metabolic adaptation should be understood as an acquired or selected consequence of successful compensation for LKB1 deficiency rather than as an inevitable direct consequence of *STK11* loss. Such selected states can exhibit altered reactive oxygen species (ROS) handling, increased resistance to ferroptosis, and greater dependence on autophagy, allowing tumor cells to persist within chronically hostile microenvironments [68,84,85,86].

At the ecosystem level, *STK11*-inactivated tumors, particularly *STK11*/LKB1-deficient LUAD, frequently exhibit an immune-cold, or immune-excluded phenotype characterized by impaired STING-dependent innate immune and interferon signaling, reduced cytotoxic T-cell infiltration, and resistance to immune-checkpoint blockade [37,71,80,81,87,88,89,90,91,92,93,94,95]. This immune resistance likely reflects both tumor-cell-intrinsic and microenvironmental mechanisms. Intrinsic defects in innate immune sensing and interferon-pathway activation can limit effective T-cell priming and recruitment, whereas loss of LKB1 activity and its associated metabolic and transcriptional rewiring can reshape the tumor microenvironment through altered cytokine and chemokine signaling, and expansion of immunosuppressive myeloid populations [81,93,94,95,96,97,98]. The resulting immune phenotype is further influenced by the cooperating genomic context; therefore, immune exclusion should not be assumed to occur uniformly in every tumor carrying an *STK11* alteration.

Collectively, somatic *STK11* inactivation can create a context-dependent evolutionary state in which metabolic, architectural, and immune constraints are progressively weakened, while the liabilities generated by loss of LKB1 signaling impose new selective pressures. Clones that acquire or engage complementary pro-grams—such as *KRAS*-driven proliferative signaling, *KEAP1* loss–NRF2-mediated redox buffering, *TP53*-associated checkpoint escape, autophagy, or other metabolic adaptations—may therefore gain a selective advantage under conditions that would otherwise restrict tumor-cell fitness.

Unlike the constitutional heterozygous state of PJS, in which higher-order tissue architecture and differentiation can remain substantially preserved, profound tumor-cell-intrinsic LKB1 deficiency within an ontogenically altered background can favor cellular plasticity, stress adaptation, immune evasion, and invasive competence, [68,71,92]. Thus, somatic *STK11* inactivation does not define a single malignant program; rather, it alters the selective landscape within which cooperating genetic and microenvironmental factors determine whether and how malignant progression occurs. This framework links *STK11* loss to adaptive persistence without excluding direct or indirect effects on proliferation and provides the basis for considering stress adaptation as a broader organizing principle in the following section.

## 5. Stress Adaptation as the Unifying Mechanism

**Figure 3:**
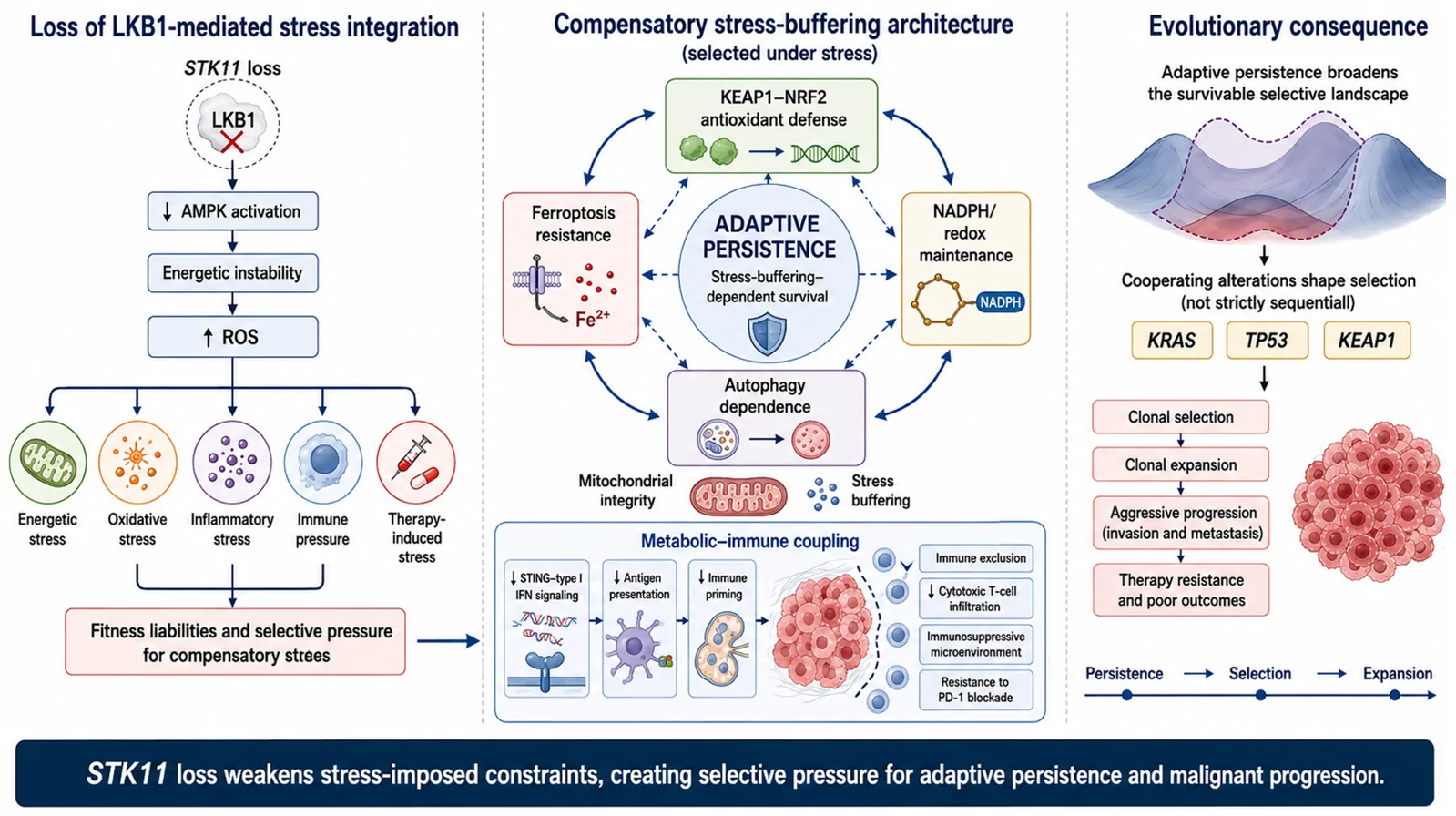
**Compensatory stress-buffering architecture and adaptive persistence following *STK11* loss.** Loss of *STK11* disrupts LKB1-mediated stress integration, creating energetic, oxidative, inflammatory, immune, and therapy-associated liabilities. Rather than directly conferring generalized stress resistance, these liabilities impose selective pressure for compensatory stress-buffering programs, which may include KEAP1–NRF2-dependent antioxidant defense, redox maintenance, autophagy dependence, mitochondrial support, and ferroptosis resistance. Metabolic–immune coupling can further impair STING–type I interferon signaling, antigen presentation, immune priming, and cytotoxic T-cell access. Together, these context-dependent programs can establish adaptive persistence supported by interconnected buffering dependencies. Cooperating alterations such as *KRAS*, *TP53*, and *KEAP1* further shape this selective landscape through distinct effects on proliferative signaling, checkpoint control, and redox homeostasis. Thus, *STK11* loss can create fitness liabilities that favor selection of compensatory states, thereby supporting clonal persistence, subsequent expansion, malignant progression, and therapeutic resistance. Solid arrows indicate directional relationships or progression; within the compensatory stress-buffering network, solid curved arrows indicate interactions among adaptive pathways, whereas dashed arrows indicate their functional association with the adaptive-persistence state. Upward and downward arrows indicate increased and decreased activity, respectively, and red X symbols indicate loss or inhibition. Colors distinguish functional modules and cellular states schematically and do not represent quantitative measurements.

Across germline and somatic contexts, we propose a unifying framework in which *STK11* deficiency reshapes cellular fitness not only through effects on proliferation but also by altering the ability of cells to integrate and withstand metabolic, oxidative, architectural, inflammatory, and immune stress. Rather than functioning solely as a classical cell-cycle gatekeeper, LKB1 serves as an integrative regulator of cellular stress responses, coordinating metabolic, redox, inflammatory, and immune homeostasis [7,23,30,68,81]. Accordingly, *STK11* loss should not be interpreted as intrinsically producing a “survival-first” state or as excluding direct effects on proliferation. Instead, impairment of LKB1 signaling relaxes selected homeostatic constraints while simultaneously generating energetic, oxidative, and structural liabilities. Cells that successfully compensate for these liabilities can acquire enhanced persistence under conditions that would otherwise restrict survival or expansion. This distinction reframes *STK11* loss not as a direct oncogenic trigger, but as a permissive alteration that reshapes how cells perceive, integrate, and respond to environmental constraints (Figure 3).

Importantly, *STK11* loss itself is not synonymous with a fully stress-adapted phenotype. Rather, LKB1 deficiency creates a permissive but vulnerable state in which continued survival depends on the successful engagement or selection of compensatory programs. These programs can include antioxidant defense, metabolic rewiring, autophagy, ferroptosis resistance, and immune-evasive mechanisms. In this model, adaptive persistence and proliferative expansion are not mutually exclusive processes; instead, stress tolerance can provide a permissive background upon which cooperating oncogenic alterations subsequently or concurrently enhance clonal fitness.

### 5.1. Molecular Rewiring of Stress Integration

Loss of LKB1 impairs AMPK activation, leading to energetic instability and elevated intracellular reactive oxygen species (ROS) [8,23,30,84]. These immediate consequences of LKB1 deficiency create metabolic and redox liabilities rather than generalized stress resistance. In cells that persist, however, selective pressure can favor coordinated rewiring of stress-buffering circuitry, including activation of KEAP1–NRF2-dependent antioxidant programs, enhanced NADPH-generating metabolic pathways, increased resistance to ferroptosis, and greater reliance on autophagy [15,72,92,99]. These adaptations are frequently reinforced by secondary genetic alterations, further stabilizing a stress-tolerant cellular state.

Importantly, these stress-buffering responses are mechanistically interconnected rather than independent. Energetic imbalance amplifies redox stress, which, in turn, increases dependence on antioxidant systems and autophagic flux to preserve mitochondrial integrity and prevent catastrophic cellular damage. *KEAP1* loss provides a particularly clear example of such cooperative adaptation: NRF2 activation enhances antioxidant and detoxification capacity, metabolic flexibility, and resistance to lipid peroxidation, thereby buffering specific oxidative liabilities associated with LKB1 deficiency [72,92]. By contrast, *TP53* loss can reduce p53-dependent checkpoint, senescence, and apoptotic barriers, thereby altering how stressed cells respond to genomic and metabolic injury [76,77]. Thus, cooperating alterations do not simply add oncogenic “hits”; they modify distinct components of the stress-response landscape. Thus, *STK11* loss does not create a collection of isolated vulnerabilities; rather, successful LKB1-deficient tumors can evolve an integrated survival architecture that buffers multiple forms of intrinsic, microenvironmental, and therapy-induced stress. The precise composition of this architecture is expected to vary according to co-mutation pattern, lineage, and tissue context.

### 5.2. Functional Consequences: Autophagy Addiction and Inflammatory Survival

A central consequence of this rewiring is increased dependence on autophagy, such that sustained autophagic flux can become important for maintaining metabolic homeostasis and buffering energetic and oxidative stress [85,86]. In parallel, loss of *STK11* reshapes inflammatory and immune signaling through metabolic–immune coupling, including alterations in cellular metabolism, redox balance, innate immune sensing, and cytokine signaling. These changes impair STING–type I interferon signaling, disrupt effective immune priming, and promote the establishment of an immunosuppressive tumor microenvironment [81,93,94,97,98]. These immune effects are particularly well established in LUAD and should not be assumed to occur uniformly across all *STK11*-altered tumors. In *KRAS*-mutant lung adenocarcinoma, *STK11* loss defines an immune-cold tumor microenvironment characterized by diminished T-cell infiltration and primary resistance to PD-1 blockade, particularly [37]. Subsequent studies further linked LKB1 deficiency to impaired antigen-processing and presentation machinery, connecting metabolic rewiring to reduced tumor immunogenicity [93,94]. Rather than representing a simple failure of immune recognition, this phenotype reflects a metabolically conditioned tumor ecosystem in which impaired innate immune activation and active immunosuppressive remodeling converge.

Consistent with this model, metabolic and redox remodeling associated with *STK11* deficiency—often reinforced by *KEAP1* co-alteration—suppresses innate immune sensing through the STING–type I interferon axis, resulting in defective immune priming and durable resistance to immunotherapy [93,97]. In redox-adapted *KEAP1/STK11*-mutant contexts, suppression of interferon signaling may extend across both DNA- and RNA-sensing pathways, including those mediated by STING and MDA5, thereby further reinforcing immune exclusion [97]. Outside LUAD, the available evidence is less extensive but supports tissue-dependent immune consequences of *STK11* perturbation; for example, *STK11* suppression has been associated with expansion of polymorphonuclear myeloid-derived suppressor cells in breast cancer [98]. These findings emphasize that the immune component of stress adaptation is strongly conditioned by tissue and co-mutational context.

Together, these features define a cellular and tissue state in which persistence under stress can be strongly favored without implying that proliferation is necessarily reduced. Inflammatory signaling, metabolic adaptation, autophagy dependence, and immune suppression constitute interdependent components of a stress-adapted survival strategy rather than isolated hallmarks of malignancy.

### 5.3. Evolutionary Implications of Stress Adaptation

From an evolutionary perspective, *STK11* loss can broaden the range of cellular states capable of persisting under adverse conditions, thereby reshaping the selective landscape in which oncogenic events operate. By altering the fitness costs associated with energetic stress, oxidative damage, architectural disruption, and immune pressure, LKB1 deficiency can create a permissive substrate in which cooperating alterations such as *KRAS*, *KEAP1*, or *TP53* provide complimentary advantages [31,71,72,74,83,84].

In this framework, there is no requirement that stress tolerance must precede proliferative signaling in a fixed temporal sequence. Rather, stress adaptation and oncogenic growth signals can arise sequentially or concurrently and can reinforce one another during clonal evolution. KRAS-driven mitogenic signaling can provide proliferative and anabolic drive, KEAP1–NRF2 activation can enhance redox and metabolic buffering, and *TP53* dysfunction can lower checkpoint and apoptotic barriers. The resulting selective advantage therefore depends on the combination of proliferative capacity and the ability to tolerate the stresses generated by tumor growth and the microenvironment.

This framework helps explain why heterozygous germline *STK11* alteration alone is generally insufficient to produce invasive malignancy, whereas profound LKB1 deficiency in an oncogenically altered somatic context can be associated with aggressive disease [49,50]. However, this distinction should not be interpreted as a universal linear progression from “survival” to “proliferation.” Instead, it reflects differences in allelic state, lineage, cooperating genetic alterations, tissue architecture, and selective pressure.

Collectively, these observations support a central evolutionary concept: *STK11* loss can promote tumor progression by relaxing stress-imposed constraints on cellular fitness while creating liabilities that favor selection for compensatory survival programs. This mechanism can operate alongside, rather than instead of, proliferative signaling. By enabling tolerance of energetic stress [83,85,86], oxidative stress [30,84,92,97], inflammatory remodeling, and immune pressure [37,80,81,93,94,97], LKB1 deficiency can contribute to an adaptive persistence state whose eventual malignant phenotype is shaped by cooperating oncogenic and microenvironmental factors.

In this framework, LKB1 functions not only as a regulator of proliferative constraints but also as a guardian of stress-imposed evolutionary constraints, whose disruption can reshape the trajectory of tumor evolution, immune adaptation, and therapeutic response.

### 5.4. Operationalizing the Stress-Adapted State: Testable Features and Predictions

For the stress-adaptation framework to be experimentally useful, it should generate testable predictions rather than serve only as a descriptive model. We therefore propose that a stress-adapted tumor state be defined not by *STK11* mutation alone or by a low proliferative index, but by the coexistence of persistent cellular stress, compensatory buffering activity, and functional dependence on those buffering systems.

At the metabolic level, candidate features include evidence of energetic disequilibrium together with compensatory metabolic rewiring, such as altered AMPK–mTOR activity, increased dependence on glutamine or other nutrient pathways, and compensatory maintenance of NADPH and redox homeostasis [72,74,83]. At the redox level, NRF2-associated transcriptional programs, antioxidant capacity, SCD1/AKR1C expression, altered lipid composition, and resistance to lipid peroxidation may indicate enhanced redox and ferroptosis buffering [72,92,99]. At the cellular-quality-control level, increased autophagic flux or selective dependence on autophagy may identify tumors in which autophagy functions as a critical metabolic survival buffer [85,100]. At the immune level, reduced STING/type I interferon signaling, impaired antigen-presentation programs, low cytotoxic T-cell infiltration, and enrichment of immunosuppressive myeloid populations may identify a stress-adapted, immune-excluded ecosystem [81,93,94,97,98].

These features should be interpreted as a multidimensional profile rather than as a single validated biomarker. A particularly important prediction of the model is functional: stress-adapted tumors should be disproportionately vulnerable when one or more compensatory buffering systems are disabled, especially when combined with an intervention that increases metabolic, oxidative, or therapy-induced stress. Thus, sensitivity to autophagy blockade, redox disruption, energetic stress, or ferroptosis induction may provide a functional readout of adaptive dependence [72,83,85,92].

Conversely, tumors dominated primarily by mitogenic signaling may exhibit less dependence on these buffering systems even when their proliferative rate is high. Importantly, stress adaptation should be dynamic rather than fixed. Longitudinal sampling before and after therapy could determine whether treatment selects for transcriptional, metabolic, or functional states with increased dependence on antioxidant defense, autophagy, ferroptosis suppression, or immune-evasive programs, thereby directly testing whether adaptive persistence emerges during therapeutic selection.

Accordingly, “stress-adapted” and “proliferation-dominant” tumors should not be regarded as mutually exclusive categories. Rather, individual tumors may occupy different positions along two partially independent dimensions—proliferative drive and stress-buffering dependence—and may shift along these dimensions during progression or therapy. This formulation makes the stress-adaptation model experimentally testable and provides a framework for identifying tumors most likely to benefit from therapies designed to exceed adaptive capacity.

## 6. Therapeutic Implications

The therapeutic logic follows directly from the stress-adaptation framework developed above: *STK11* loss can simultaneously relax growth-regulatory constraints and create metabolic, oxidative, and immune liabilities that select for compensatory survival programs. Consequently, therapeutic vulnerability may arise not only from oncogenic signaling itself, but also from the acquired dependencies that allow LKB1-deficient tumor cells to tolerate these stresses. The most durable therapeutic leverage points may therefore include both conventional tumor-driving pathways and the compensatory stress-buffering systems on which stress-adapted LKB1-deficient tumors become dependent. Rather than simply increasing cytotoxic pressure along a single axis, an important therapeutic objective may be to identify and disrupt these context-specific adaptive dependencies, particularly in tumors in which multiple buffering mechanisms cooperate to maintain fitness.

### 6.1. Why STK11-Inactivated/LKB1-Deficient Tumors Resist Standard Modalities

#### 6.1.1. Immune Checkpoint Blockade (ICB): Immune Exclusion and Context-Dependent Resistance

Clinical meta-analyses and cohort studies have associated *STK11* alterations in NSCLC with reduced benefit from immune checkpoint blockade, lower PD-L1 expression, and adverse clinical outcomes, although the magnitude and independence of these associations vary according to co-mutational and clinical context [101]. Across LUAD cohorts, *STK11* alterations—particularly in the context of *KRAS* co-mutation—are consistently associated with primary resistance to PD-1/PD-L1 blockade, defining a distinct immune-excluded (“immune-cold”) tumor ecosystem [37,80,95]. These tumors exhibit reduced T-cell–inflamed transcriptional signatures, diminished cytotoxic lymphocyte infiltration, and impaired interferon responses, collectively providing a biological basis for reduced sensitivity to immunotherapy in a subset of *STK11*/LKB1-deficient tumors.

Mechanistically, LKB1 deficiency and associated co-mutational states can impair innate immune sensing and interferon signaling. In particular, *STK11*/LKB1 loss has been linked to suppression of STING–type I interferon signaling, whereas *KEAP1/STK11*-associated redox remodeling can further suppress innate immune sensing pathways, including STING- and MDA5-mediated signaling. These alterations can result in attenuate type I interferon programs, impair immune priming and contribute to T-cell exclusion [93,94,97,102]. This redox-dependent repression of antiviral and inflammatory signaling establishes immune evasion as an intrinsic ecological feature of LKB1-deficient tumors rather than merely a secondary consequence of disease progression. Thus, immune resistance in *STK11*-altered LUAD is better understood as a context-dependent phenotype generated through the interaction of tumor-cell-intrinsic LKB1 deficiency, redox and metabolic state, cooperating genomic alterations, and microenvironmental remodeling, rather than as an invariant consequence of *STK11* mutation alone.

#### 6.1.2. Metabolic Stress and Therapy-Induced Stress: Adaptive Dependencies Rather than “Built-In” Buffering

Loss of LKB1 disrupts AMPK–mTOR-dependent energy sensing and rewires broader metabolic programs, fundamentally reshaping how tumor cells respond to energetic stress and altering their dependence on mitochondrial and nutrient-derived energy sources [74,82]. Importantly, these effects do not imply that LKB1-deficient cells possess an intrinsic or “built-in” capacity to tolerate metabolic stress. As discussed above, acute impairment of LKB1 signaling can generate energetic and oxidative liabilities. Tumor cells that persist despite these liabilities may therefore become increasingly dependent on alternative metabolic and cytoprotective pathways, creating context-specific therapeutic vulnerabilities.

Consistent with this model, experimental studies have identified selective sensitivities of LKB1-deficient tumors to interventions that intensify energetic or mitochondrial stress or disrupt compensatory metabolism. Pharmacological inhibition of mitochondrial complex I with phenformin induces a profound energetic crisis and cell death in *KRAS*-driven, *Stk11*-deficient lung tumors, revealing a genotype-specific sensitivity to metabolic stress [31]. Similarly, approaches that intensify energetic stress selectively impair the survival of LKB1-deficient NSCLC cells [83], whereas LKB1-deficient tumors may display increased vulnerability to glucose-deprivation-induced cell death [103]. Together, these findings indicate that *STK11* loss does not confer generalized metabolic robustness; instead, it creates conditional stress tolerance coupled with heightened dependence on specific compensatory pathways that can be therapeutically exploited.

#### 6.1.3. Radiation and DNA-Damaging Therapy: Stress Tolerance and DNA-Repair Vulnerability

LKB1-deficient NSCLC tumors exhibit relative resistance to radiotherapy through KEAP1–NRF2-dependent redox buffering, indicating that ionizing radiation imposes substantial oxidative and metabolic stress that can be buffered in appropriately adapted tumor contexts rather than uniformly translated into cell death [15]. Radiotherapy generates not only DNA damage but also oxidative and metabolic stress, and the cellular outcome therefore depends on the balance between the magnitude of therapy-induced injury and the capacity of tumor cells to engage compensatory stress-response programs. In this context, resistance to radiation and chemotherapy may arises not solely from altered DNA-damage-response capacity but also from selected or co-mutation-dependent stress-buffering programs that mitigate therapy-induced oxidative, metabolic, and mitochondrial stress.

LKB1 also participates directly in the DNA-damage response, providing an additional link between *STK11* status and sensitivity to genotoxic therapy. Wang et al. showed that LKB1 is recruited to sites of DNA damage and contributes to DNA-damage signaling and repair; LKB1 deficiency impaired homologous recombination and increased sensitivity to PARP inhibition [104]. These findings indicate that *STK11* loss can generate DNA-repair liabilities even while other adaptive programs promote tolerance of therapy-induced stress. Thus, the response of LKB1-deficient tumors to radiation or DNA-damaging therapies should not be viewed as uniformly resistant; rather, it reflects the balance between specific repair defects and the compensatory mechanisms that permit stressed cells to survive.

This duality has therapeutic implications: radiation and cytotoxic therapies may serve not only as direct cytotoxic agents but also as stress-amplifying components of rational combination regimens, whereas DNA-repair defects associated with LKB1 loss may create additional opportunities for synthetic-lethal strategies such as PARP inhibition [104].

### 6.2. Where the Vulnerabilities Lie

#### 6.2.1. Redox Collapse: Turning Adaptation into Liability

If LKB1-deficient tumors develop enhanced dependence on redox-buffering programs to tolerate elevated reactive oxygen species (ROS), the therapeutic objective is not necessarily to reduce ROS but to push the system beyond its redox-buffering capacity, thereby inducing catastrophic oxidative damage and potentially disrupting redox-coupled immune suppression [30]. This strategy is particularly relevant in tumors with concurrent *KEAP1* loss, in which NRF2 activation strengthens antioxidant defense, detoxification, and metabolic flexibility and can therefore buffer oxidative liabilities associate with LKB1 deficiency [72,92]. Thus, the therapeutic relevance of redox targeting is expected to depend substantially on co-mutational context rather than on *STK11* status alone.

In *KRAS*-mutant lung cancers, *STK11* loss promotes epigenetic silencing of the *TMEM173* gene encoding STING, thereby preventing pathological activation of IRF3–STAT1 signaling driven by mitochondrial dysfunction and cytoplasmic DNA accumulation [94]. This mechanism illustrates how suppression of excessive innate immune activation can support tumor cell fitness while simultaneously creating a potentially exploitable immune signaling vulnerability. Further supporting this framework, transient induction of genomic instability and innate immune activation can overwhelm adaptive buffering, restore T-cell infiltration, and sensitize tumors to PD-1 blockade [105].

Redox adaptation therefore represents both a survival mechanism and a potential point of fragility. In *STK11/KEAP1*-coaltered tumors, interventions that increase mitochondrial or oxidative stress while simultaneously restricting NRF2-dependent antioxidant capacity may be particularly effective because they remove the buffering system on which the adapted state has become dependent. These findings support combination strategies in which one component increases oxidative or mitochondrial stress while another disables compensatory antioxidant or stress-response pathways, thereby potentially exceeding the redox-buffering capacity of the tumor and producing a nonlinear loss of cellular fitness [72].

#### 6.2.2. Autophagy Blockade: Removing a Central Adaptive Survival Buffer

Together with redox buffering, autophagy constitutes a complementary stress-dissipation axis. LKB1-deficient tumors can become dependent on autophagy as an adaptive survival program that mitigates energetic, oxidative, and therapy-induced stress. Therapeutic interventions may induce cytoprotective autophagy in LKB1-deficient contexts; inhibiting this response may shift adaptation toward cell death or loss of fitness, thereby restraining tumor progression [106]. This relationship is context-dependent and illustrates the dual role of the LKB1–AMPK axis in cancer. In LKB1-proficient cells, LKB1–AMPK–ULK1 signaling can promote autophagy as part of the physiological response to energetic or therapy-induced stress [107]. Loss of LKB1 therefore does not intrinsically predict increased autophagic activity. Rather, established LKB1 deficient tumors that successfully persist despite impaired canonical LKB1 signaling may engage alternative autophagy-regulatory circuits and become selectively dependent on autophagic flux for survival. For example, circHIPK3–STAT3–AMPK signaling has been reported to enhance autophagic flux in *STK11*-mutant lung cancer, supporting metabolic adaptation and tumor-cell survival [100]. Although circHIPK3 may regulate autophagy differently across biological systems, available evidence in this context supports a proautophagic role. Sustained stress-buffering autophagy may therefore contribute to therapeutic resistance and adverse clinical outcomes in established tumors.

Beyond tumor-intrinsic survival, autophagy also modulates tumor–immune interactions. Experimental targeting of autophagy, combined with remodeling of the tumor microenvironment, has been shown to enhance responsiveness to ICB in *STK11*-mutant lung cancer models [108]. More broadly, autophagy enables tumor cells to withstand diverse therapeutic stresses in both *STK11*-altered and *STK11*-wild-type settings, reinforcing its role as a general adaptive mechanism rather than an exclusively mutation-specific feature [85,86].

Collectively, these findings position autophagy as a context-dependent adaptive dependency rather than a constitutive consequence of *STK11* loss and support its therapeutic targeting primarily as a sensitization strategy rather than as a stand-alone cytotoxic intervention.

#### 6.2.3. Ferroptosis: Exploiting Lipid-Peroxidation Fragility in Redox-Rewired Tumors

Redox-rewired tumors frequently evade ferroptosis to tolerate chronic oxidative stress, creating a vulnerability linked to lipid-metabolic remodeling. However, the mechanistic basis of this resistance is not uniform across *STK11*-altered tumors and appears to be strongly influenced by cooperating alterations, particularly *KEAP1* loss and NRF2 activation.

In *STK11*/KEAP1coaltered lung cancers, NRF2-driven antioxidant adaptation is coupled to changes in lipid metabolism that reduce susceptibility to iron-dependent lipid peroxidation. Concurrent loss of *STK11* and *KEAP1* promotes increased expression of SCD1 and AKR1C-family protein expression, together with increased monounsaturated fatty acid (MUFA) production [92]. By shifting membrane-lipid composition toward MUFAs, these cells reduce the abundance or peroxidizability of lipid species that would otherwise propagate ferroptotic damage. AKR1C-family enzymes provide an additional protective layer by detoxifying reactive lipid-peroxidation products. The resulting phenotype is therefore not simply “low ROS,” but an actively maintained state in which antioxidant signaling and membrane-lipid remodeling cooperate to suppress ferroptotic execution.

This adaptation creates a corresponding dependency. Concurrent *STK11/KEAP1* loss renders tumor cells selectively dependent on lipid-desaturation pathways, including SCD1, such that disruption of this protective machinery can restore lipid-peroxidation sensitivity [92].

Independent analyses further indicate that *STK11* alteration itself can promote MUFA synthesis and preserve mitochondrial integrity [99]. These observations suggest that *STK11* loss and *KEAP1* loss–NRF2 activation can converge on ferroptosis protection through overlapping but non-identical mechanisms, with the strongest evidence currently derived from LUAD.

Co-mutational context is therefore likely to determine the depth of ferroptosis resistance and the most effective therapeutic entry point. *STK11/KEAP1*-coaltered tumors may be particularly dependent on NRF2-driven antioxidant and lipid-protective programs, whereas *STK11*-altered tumors lacking KEAP1 activation may rely on different combinations of metabolic adaptation, lipid desaturation, glutathione metabolism, or mitochondrial protection. This distinction is important therapeutically because sensitivity to ferroptosis induction should not be inferred from *STK11* mutation status alone.

Tissue context may also modify ferroptosis susceptibility. The current mechanistic evidence for *STK11*-associated ferroptosis resistance is concentrated in LUAD, and comparable dependencies have not yet been established to the same extent in pancreatic, cervical, endometrial, melanoma, or other *STK11*-altered malignancies. Because the current mechanistic evidence is concentrated in LUAD, whether comparable ferroptosis-protective dependencies operate in pancreatic, cervical, endometrial, melanoma, or other *STK11*-altered malignancies remains to be established. Thus, prospective functional profiling will be required before ferroptosis-directed strategies can be generalized across tumor types.

Ferroptosis also intersects with antitumor immunity, although this relationship is context-dependent. Activated CD8^+^ T cells can promote tumor-cell ferroptosis through IFNγ-mediated suppression of the SLC3A2/SLC7A11 cystine-transport system, and ferroptosis can contribute to the antitumor efficacy of immunotherapy [109]. However, ferroptotic cell death is not uniformly immunogenic; its effects on antitumor immunity depend on the stage and extent of ferroptosis, tumor context, and the susceptibility of immune-cell populations to lipid peroxidation [110]. Thus, whether ferroptosis induction can therapeutically cooperate with immune reactivation in LKB1-deficient tumors remains an important question for future investigation. Together, these findings establish ferroptosis resistance as an active, metabolically maintained adaptation and support ferroptosis re-engagement—through inhibition of lipid desaturation, disruption of antioxidant protection, or direct induction of ferroptosis—as a rational strategy for exploiting latent lipid-peroxidation fragility. The most promising applications are likely to require biomarker guided selection based on *STK11/KEAP1* status, NRF2 activity, lipid-metabolic state, and functional ferroptosis sensitivity rather than *STK11* genotype alone.

#### 6.2.4. Synthetic Stress Overload: Engineered Ecological Failure

LKB1-deficient tumors can occupy a stress-adapted equilibrium sustained by coordinated buffering across metabolic, redox, autophagic, ferroptosis protective, and immune-regulatory programs. As emphasized above, this state is not an automatic consequence of *STK11* loss but represents successful compensation for the energetic, oxidative, and structural liabilities created by impaired LKB1 signaling. Perturbation of a single axis may be accommodated through compensatory plasticity; however, regimens deliberately designed to impose simultaneous, mechanistically distinct stressors may overwhelm this adaptive capacity. In this framework, therapeutic efficacy arises not simply from intensifying a single insult but from disrupting multiple conditions required to maintain stress tolerance. Representative therapeutic strategies follow a common logic. First, combining energetic stress with autophagy blockade may convert cytostatic pressure into apoptotic or immunogenic collapse by removing a critical survival buffer [85,86,106]; Second, elevating ROS while inhibiting antioxidant or lipid-protective pathways may dismantle redox tolerance and expose latent ferroptosis sensitivity [88]. Third, combining radiation-induced DNA damage with disruption of redox defenses or ferroptosis induction may integrate genotoxic, oxidative, and lipid-peroxidation stress, potentially extending therapeutic pressure from tumor-intrinsic survival pathways to the tumor–immune interface [15,30,92].

The rationale for such combinations is therefore not simply “more therapy,” but mechanistic complementarity: one intervention generates a defined stress while another removes the compensatory pathway required to tolerate it. This distinction is important because indiscriminate multi-drug treatment may increase toxicity without necessarily exceeding tumor adaptive capacity. Rational combinations should instead be selected according to the dominant buffering dependencies present in a given genetic and tissue context.

This framework also predicts that therapeutic combinations may need to differ among co-mutational states. For example, *STK11/KEAP1*-coaltered tumors may be particularly suited to strategies that combine oxidative or metabolic stress with disruption of NRF2-associated redox or ferroptosis-protective pathways, whereas *TP53* co-alteration may further influence therapeutic response through altered checkpoint and apoptotic signaling, although this interaction remains less well defined. Thus, the concept of engineered ecological failure is inherently genotype- and state-dependent rather than a universal combination template.

Together, these observations suggest that durable therapeutic responses in LKB1-deficient cancers are unlikely to arise solely from intensified monotherapies and may instead require rationally layered combinations that exceed the limits of adaptive stress tolerance. Within this framework proposed here, the goal is to convert acquired buffering dependence into therapeutic vulnerability while avoiding the assumption that every LKB1-deficient tumor shares the same adaptive architecture.

**Figure 4:**
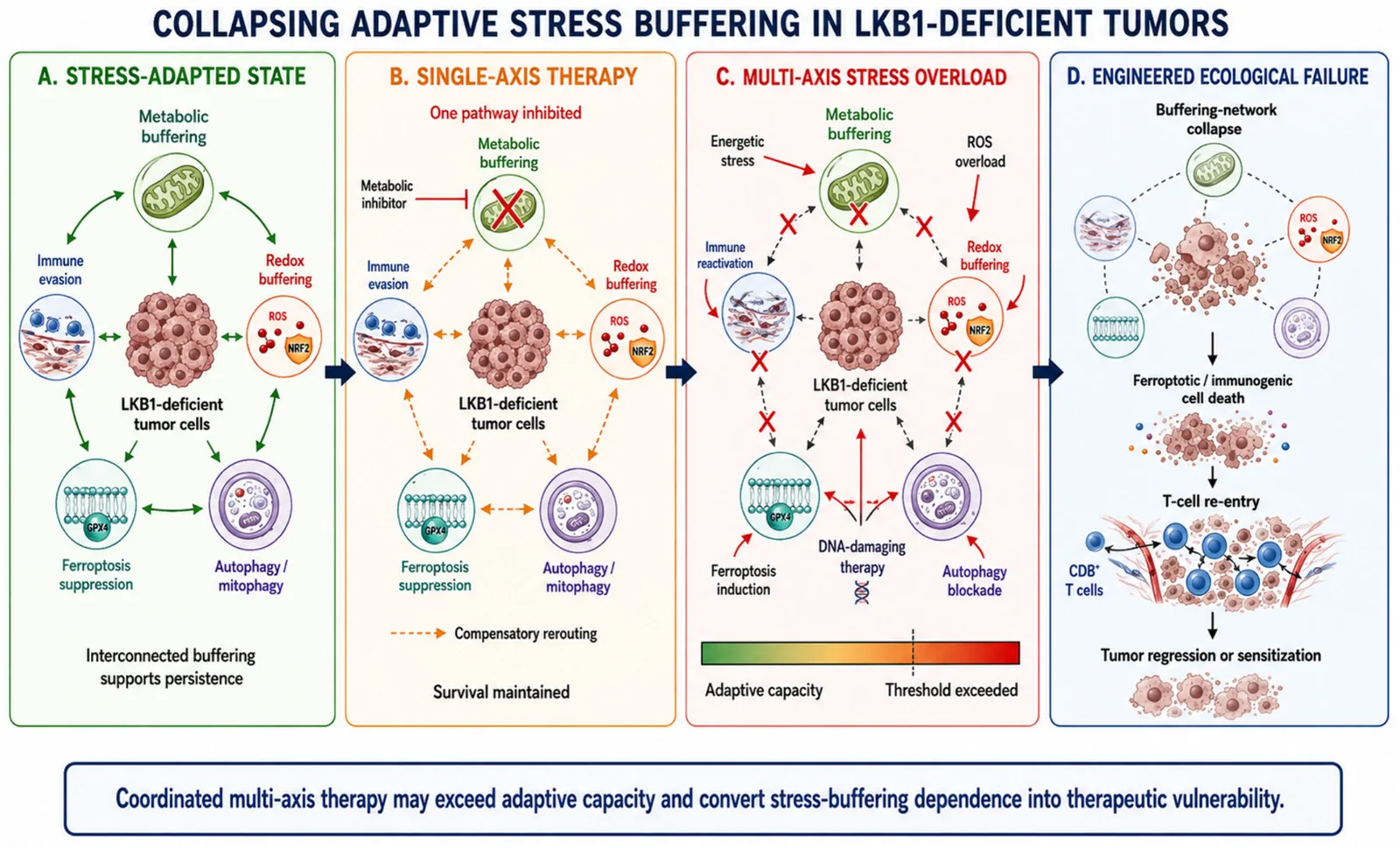
**Therapeutic collapse of adaptive stress buffering in LKB1-deficient tumors.** (**A**) Stress-adapted LKB1-deficient tumor cells can maintain persistence through interconnected metabolic and redox buffering, autophagy and mitophagy, ferroptosis suppression, and immune-evasive programs. (**B**) Inhibition of a single pathway may trigger compensatory rerouting through remaining buffering networks, allowing tumor-cell survival to be maintained. (**C**) Coordinated multi-axis therapy combines complementary interventions—including energetic stress, redox disruption, autophagy blockade, ferroptosis induction, DNA-damaging therapy, and immune reactivation—to disrupt multiple compensatory pathways. When cumulative stress exceeds adaptive capacity, these buffering networks may no longer preserve tumor-cell fitness. (**D**) The resulting “engineered ecological failure” may promote buffering-network collapse, ferroptotic and/or immunogenic tumor-cell death, restoration of CD8^+^ T-cell access, and tumor regression or therapeutic sensitization. Thus, rational multi-axis treatment may exploit stress-buffering dependencies in *STK11*-inactivated, LKB1-deficient tumors by converting adaptive persistence into therapeutic vulnerability. Solid arrows indicate directional relationships or progression, whereas dashed arrows indicate compensatory or functional interactions among stress-buffering pathways. T-bar symbols indicate pathway inhibition, and red X symbols indicate disruption or collapse of the indicated buffering mechanisms. The green-to-red gradient represents increasing stress burden relative to adaptive capacity, with red indicating that the adaptive threshold has been exceeded. Other colors are used schematically to distinguish functional pathways and cellular states.

By reframing therapeutic resistance as a consequence of successful stress adaptation, this perspective identifies stress-buffering systems as actionable vulnerabilities rather than intractable barriers. Accordingly, combination strategies designed to exceed the adaptive capacity of tumor cells may offer a rational approach for targeting LKB1-deficient cancers, although specific combinations will require validation according to co-mutation status, tissue lineage, and functional evidence of adaptive dependence (Figure 4).

## 7. Outlook: Stress Adaptation as a General Principle in Cancer Therapy Design

Although this Review focuses on *STK11*, the stress-adaptation framework may have broader implications for cancer biology and therapy. A central principle emerging from LKB1-deficient tumors is that loss of a homeostatic regulator does not necessarily confer stress resistance directly; rather, it can reshape the selective landscape in which compensatory states capable of sustaining survival are favored. KEAP1 co-inactivation provides a particularly relevant example in LKB1-deficient lung cancer, where NRF2 activation can reinforce antioxidant and metabolic buffering. However, whether analogous stress-adaptive trajectories operate across other genetic backgrounds and tumor types remains to be established. Thus, the framework proposed here should be viewed as a testable model rather than a universal property of tumor-suppressor loss.

Viewing stress buffering as a selectable and evolvable trait exposes an important limitation of conventional monotherapies. Therapeutic strategies that intensify a single stress axis, whether metabolic, genotoxic, oxidative, or immune-mediated, may be accommodated through compensatory plasticity within interconnected buffering networks. As a result, therapeutic pressure can select for increasingly stress-tolerant cellular states rather than inducing durable tumor collapse. This perspective supports mechanism-based combinations in which one intervention imposes a defined stress while another disables the compensatory pathway required to tolerate that stress, thereby pushing tumor cells beyond their adaptive capacity.

Translating this framework into clinically useful strategies will require biomarkers that distinguish genotype from functional stress-adaptive state. *STK11* alteration alone is unlikely to provide sufficient predictive resolution because adaptive phenotypes are shaped by allelic status, functional class of the alteration, cooperating mutations, tissue lineage, and treatment history. Candidate indicators may therefore include co-mutational context, pathway-level transcriptional signatures, metabolic and redox states, autophagic dependence, ferroptosis sensitivity, and features of the immune microenvironment. Longitudinal assessment may be particularly important because therapeutic pressure can dynamically reshape these dependencies.

More broadly, this perspective encourages a shift in therapeutic logic—from targeting oncogenic signaling pathways in isolation toward integrating oncogenic drivers with the adaptive systems that sustain tumor fitness under stress. In the conceptual model advanced here, proliferative signaling and stress adaptation are complementary rather than competing determinants of malignancy: oncogenic pathways promote expansion, whereas stress-buffering programs determine whether that expansion can be maintained under energetic, oxidative, immune, and therapy-induced constraints.

Cancer progression and treatment response are shaped not solely by pathway activation, but also by the ability of tumor cells to negotiate energetic, redox, inflammatory, and structural constraints imposed by their microenvironment. Importantly, this framework is intended to complement rather than replace oncogene-directed therapy: proliferative signaling determines important components of tumor fitness, whereas stress-buffering programs determine whether that fitness can be maintained under hostile conditions. Integrating these dimensions may therefore provide a more complete basis for therapeutic design and improve the durability of therapeutic responses, particularly in stress-adapted malignancies.

Accordingly, we propose “engineered ecological failure” as a testable therapeutic framework in which a tumor-specific stress is intensified while the adaptive mechanism required to buffer that stress is simultaneously disrupted. In *STK11*-inactivated cancers, this strategy may convert dependencies on metabolic plasticity, antioxidant defense, autophagy, ferroptosis suppression, or immune escape into therapeutic liabilities. Prospective experimental and clinical studies will be required to determine which combinations, biomarkers, and tumor contexts can translate this conceptual framework into durable therapeutic benefit.

## 8. Conclusions

*STK11*/LKB1 loss in cancer is best understood not simply as the removal of a proliferative brake, but as a disruption of the homeostatic systems that couple cellular growth and survival to metabolic, oxidative, architectural, and immune constraints. The biological consequences of this disruption are strongly context dependent. Heterozygous germline *STK11* alteration in Peutz–Jeghers syndrome establishes a cancer-predisposed but often architecturally constrained state, whereas profound LKB1 deficiency in established tumors can interact with tissue-specific and cooperating genetic alterations to promote malignant progression, immune evasion, and therapeutic resistance.

Importantly, LKB1 deficiency does not intrinsically confer generalized stress resistance. Instead, loss of normal stress integration creates energetic, redox, structural, and immune liabilities that impose selective pressure for compensatory states capable of sustaining cellular fitness. Successful tumors may therefore become increasingly dependent on interconnected buffering mechanisms, including metabolic rewiring, antioxidant defense, autophagy, ferroptosis suppression, and immune-evasive programs. These dependencies provide a conceptual link between the biological consequences of *STK11* loss and its therapeutic vulnerabilities.

This framework suggests that *STK11* genotype alone is unlikely to adequately predict tumor behavior or therapeutic response. Integrating allelic and functional LKB1 status with co-mutational context, tissue lineage, and the specific stress-buffering dependencies of individual tumors may provide a more informative basis for patient stratification and therapeutic design. Ultimately, rational strategies that simultaneously increase defined cellular stresses and disable the compensatory mechanisms required to tolerate them may convert adaptive persistence into therapeutic vulnerability. Testing this model across tumor types and defining biomarkers of functional stress adaptation will be essential for translating the biology of LKB1 deficiency into more durable therapeutic strategies.

## Figures and Tables

**Table 1:** Genetic and functional heterogeneity of *STK11* alteration.

*STK11* Genetic/Functional State	Typical Context	Expected LKB1 Status	Biological Interpretation	Representative References
Heterozygous germline pathogenic variant	Constitutional PJS state	One pathogenic allele; one WT allele initially retained	Establishes inherited cancer predisposition; not equivalent to complete LKB1 loss	[5,6,45,46]
Functional haploinsufficiency	PJS-associated tissue growth; *Stk11*^+/−^ experimental models	Reduced effective LKB1 dosage despite retention of WT allele	Partial dosage reduction can be sufficient for hamartomatous growth without obligatory LOH	[49,50,58]
Germline variant + somatic second hit	Subset of PJS polyps/neoplasms and PJS-associated cancers	Further impairment/loss of WT allele through LOH or somatic mutation	Can contribute to progression, but is not universally required for hamartoma formation	[20,56,57]
Somatic monoallelic or functionally partial alteration	Sporadic cancers	Variable residual LKB1 activity	Functional consequences depend on mutation type and residual protein function	[17,59]
Somatic biallelic *STK11* inactivation	Established sporadic cancers, particularly well characterized in LUAD	Profound or complete LKB1 deficiency	Strong disruption of LKB1-dependent homeostasis; biological consequences shaped by co-mutations and tissue context	[9,14,59]
Functionally heterogeneous missense variants	Sporadic cancers	Variant-dependent kinase activity, localization, stability, or signaling	*STK11* mutation should not automatically be equated with complete LKB1 loss	[17,59]

Abbreviations: LOH, loss of heterozygosity; PJS, Peutz–Jeghers syndrome; WT, wild-type; LUAD, lung adenocarcinoma.

## Data Availability

No new data were created or analyzed in this study. Data sharing is not applicable to this article.

## Abbreviations

The following abbreviations are used in this manuscript:

STK11	Serine/threonine kinase 11
LKB1	liver kinase B1
PJS	Peutz–Jeghers syndrome
NSCLC	Non–Small Cell Lung Cancer
LUAD	Lung Adenocarcinoma
KRAS	Kirsten Rat Sarcoma Viral Oncogene Homolog
TP53	Tumor Protein p53
NF1	Neurofibromin 1
KEAP1	Kelch-like ECH-associated Protein 1
NRF2	Nuclear Factor, Erythroid 2–Like 2
AMPK	AMP-Activated Protein Kinase
mTOR	Mechanistic Target of Rapamycin
SIRT1	Sirtuin 1
ULK1	Unc-51 Like Autophagy Activating Kinase 1
STING	Stimulator of Interferon Genes
IRF3	Interferon Regulatory Factor 3
STAT1	Signal Transducer and Activator of Transcription 1
PD-1	Programmed Cell Death Protein 1
PD-L1	Programmed Death-Ligand 1
NF-κB	Nuclear Factor Kappa B
SCD1	Stearoyl-CoA Desaturase 1
AKR1C	Aldo–Keto Reductase Family 1 Member C
CDC42	Cell Division Cycle 42
ROS	Reactive Oxygen Species
NADPH	Nicotinamide Adenine Dinucleotide Phosphate (Reduced Form)
MUFAs	Monounsaturated Fatty Acids
ICB	Immune Checkpoint Blockade
